# Oscillatory Potentials in Achromatopsia as a Tool for Understanding Cone Retinal Functions

**DOI:** 10.3390/ijms222312717

**Published:** 2021-11-24

**Authors:** Giulia Righetti, Melanie Kempf, Christoph Braun, Ronja Jung, Susanne Kohl, Bernd Wissinger, Eberhart Zrenner, Katarina Stingl, Krunoslav Stingl

**Affiliations:** 1 Center for Ophthalmology, University Eye Hospital, University of Tübingen, 72076 Tübingen, Germany; melanie.kempf@med.uni-tuebingen.de (M.K.); ronja.jung@med.uni-tuebingen.de (R.J.); katarina.stingl@med.uni-tuebingen.de (K.S.); krunoslav.stingl@med.uni-tuebingen.de (K.S.); 2 Center for Rare Eye Diseases, University of Tübingen, 72076 Tübingen, Germany; ez@uni-tuebingen.de; 3 MEG-Center, University of Tübingen, 72076 Tübingen, Germany; christoph.braun@uni-tuebingen.de; 4 CIMeC, Center for Mind/Brain Science, University of Trento, 38123 Trento, Italy; 5 Molecular Genetics Laboratory, Center for Ophthalmology, Institute for Ophthalmic Research, University of Tübingen, 72076 Tübingen, Germany; susanne.kohl@med.uni-tuebingen.de (S.K.); bernd.wissinger@med.uni-tuebingen.de (B.W.); 6 Center for Ophthalmology, Institute for Ophthalmic Research, University of Tübingen, 72076 Tübingen, Germany; 7 Werner Reichardt Centre for Integrative Neuroscience (CIN), University of Tübingen, 72076 Tübingen, Germany

**Keywords:** ERG, achromatopsia, oscillatory potentials, cone functions, Morlet wavelet transform, time-frequency analysis

## Abstract

Achromatopsia (ACHM) is an inherited autosomal recessive disease lacking cone photoreceptors functions. In this study, we characterize the time-frequency representation of the full-field electroretinogram (ffERG) component oscillatory potentials (OPs), to investigate the connections between photoreceptors and the inner retinal network using ACHM as a model. Time-frequency characterization of OPs was extracted from 52 controls and 41 achromat individuals. The stimulation via ffERG was delivered under dark-adaptation (DA, 3.0 and 10.0 cd·s·m^−2^) to assess mixed rod-cone responses. The ffERG signal was subsequently analyzed using a continuous complex Morlet transform. Time-frequency maps of both DA conditions show the characterization of OPs, disclosing in both groups two distinct time-frequency windows (~70–100 Hz and >100 Hz) within 50 ms. Our main result indicates a significant cluster (*p* < 0.05) in both conditions of reduced relative power (dB) in ACHM people compared to controls, mainly at the time-frequency window >100 Hz. These results suggest that the strongly reduced but not absent activity of OPs above 100 Hz is mostly driven by cones and only in small part by rods. Thus, the lack of cone modulation of OPs gives important insights into interactions between photoreceptors and the inner retinal network and can be used as a biomarker for monitoring cone connection to the inner retina.

## 1. Introduction

Achromatopsia (ACHM) is a rare autosomal recessive inherited disease with an incidence of 1 in 30,000 [1,2,3]. ACHM is due to congenital dysfunction of the cone photoreceptors and biallelic mutations in six different genes (i.e., *CNGA3*, *CNGB3*, *GNAT2*, *PDE6H*, *PDE6C*, and *ATF6*) that are responsible for >90% of the cases [4]. Individuals affected by ACHM (achromats) are clinically characterized by poor visual acuity, absence of color perception, nystagmus, photophobia, and central scotoma [1,5,6]. Electrophysiological recordings show that achromats have notably reduced or unmeasurable cone responses following full-field photopic stimulation, and usually near to normal responses of rods in the scotopic assessment [7,8]. However, previous studies have also reported a reduction in a- and b-wave amplitude in rod responses under dark-adapted assessments [9,10].

Full-field electroretinography (ffERG) is a powerful non-invasive tool that allows for the assessment of retinal function in response to stimuli of different temporal and spectral luminance characteristics. The ffERG consists of a sequence of several components. The major light-driven components are represented by a-wave, b-wave, oscillatory potentials (OPs), and flicker responses of the standardized ffERG [11]. In all conditions, the a-wave is elicited by the hyperpolarization of the photoreceptors in the outer nuclear layer, while the subsequent b-wave reflects the depolarization of the ON-bipolar cells in the inner retinal layer in conjunction with the Müller cells [12,13,14]. Moreover, filtering the signal makes it possible to extract the OPs, which are rhythmic fast oscillations identified in high-frequency bands (75–300 Hz), raising between the a-wave and the ascending phase of the b-wave [11].

Some theories suggest that OPs may originate in the inner nuclear layer [15,16] and arise from the interaction of excitatory and inhibitory circuitry provided by bipolar, amacrine, and ganglion cells elicited by both types of photoreceptors [17,18,19]. In finding the source of these fast oscillations, previous studies have focused on their characterization over time and in the frequency domain. Regarding the temporal domain (e.g., temporal sequence and amplitude behavior to stimulus interval [20]), the presence and amplitude of peaks were observed ranging from four to six in total number [21,22,23]. On the frequency domain, OPs were differentiated when elicited under dark or light adaptation. Specifically, the peak average in the frequency spectrum occurs at lower frequencies under light adaptation in respect to dark-adapted stimulations [24,25,26].

Furthermore, in studies with patients with cone or rod dysfunction, it has been assumed that these peaks reflect differing involvement of photoreceptors, whereby cones and rods contribute more to early and late OPs’ subcomponents, respectively [27,28], or to the OFF-response [20]. Studies have also reported that it is possible to modulate these subcomponents in OPs using a pharmacological intervention. Dong and co-workers (2004) consider that multiple generators in the inner retina are involved in the origin of the OPs, and report that the subcomponents of OPs can be selectively manipulated [29]. In their study, they suggest that the early OP components are photoreceptor-driven and late OP peaks are produced by inner retinal generators. Regarding intermediate components, pharmacological agents that block specific transmitter receptors or voltage-gated channels suggest intermediate OP peaks are primarily generated by action-potential-independent synaptic interactions. Additionally, the generation of late components occurs from action-potential-dependent interactions between third-order neurons in the ON pathway. However, the origin and function of OPs are still an ongoing cause for debate.

The standard approach in characterizing OPs is to perform filtering of ffERG traces to exclude components such as a-wave and b-wave having low-frequency bands. The use of the Fourier transform allows for visualization of the frequencies that comprise the signal, and for easy detection of the range of high frequencies that correspond to the OPs. The use of band-pass filtering to select the frequency range of the OPs, therefore, allows the observation of their dynamics over time and the mining of their peaks and amplitudes. Another method, known as continuous wavelet transform, consists in extracting simultaneously both time and frequency information through the convolution of a kernel (wavelet) on the target trace. The characterization of the ffERG signal in the time and frequency domains has emerged as a suited method for assessing retinal components in normal and diseased retinae [30,31,32,33].

The purpose of this study is to investigate the characteristics of retinal components elicited by light stimulation after dark adaptation in standard full-field ERG recordings in healthy controls and ACHM individuals using a complex Morlet wavelet transform. This approach allows for the comparison of the two groups in the time and frequency domains to better understand the underlying dynamics of OPs.

## 2. Results

### 2.1. Electrophysiological Results

**Figure 1:**
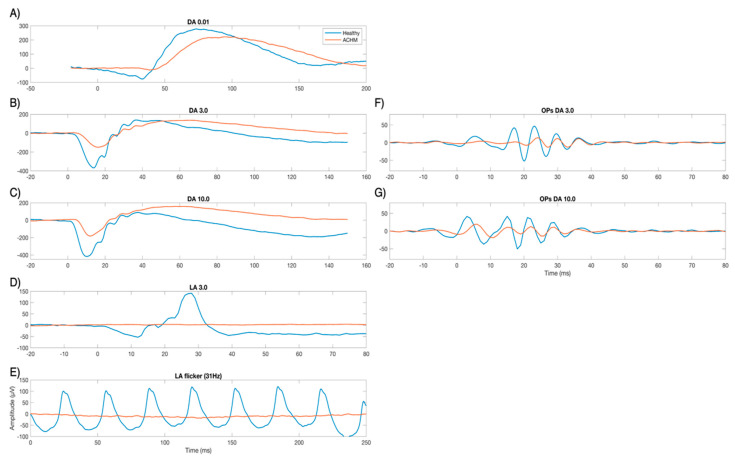
Full−field ERG according to the ISCEV (International Society for Electrophysiology of Vision) standards. Representative cases of one healthy control and one ACHM individual marked with blue and orange lines, respectively. On the left, the plots show standard responses of dark-adapted (DA) 0.01 (**A**), DA 3.0 (**B**), DA 10.0 (**C**), and light−adapted (LA) 3.0 (**D**) and LA flicker at 31 Hz (**E**). On the right panel, the plots (**F**,**G**) show the filtered traces (75–300 Hz) OPs of DA 3.0 and DA 10.0, respectively.

Figure 1 shows two typical cases of ERG traces of a healthy subject and an ACHM subject. For illustrative purposes, clinical assessments of the standard full-field ERG are shown from scotopic to photopic stimulation. As can be observed, in the dark-adapted 0.01 cd·s·m^−2^ condition, the ACHM subject shows a response close to normal. With increasing intensity and shifting toward the photopic conditions, stimulation leads to a mixed response of cones and rods (DA 3.0 and DA 10.0). Regarding the latter, the lack of cone functionality in ACHM subjects, clearly visible in photopic evaluations (LA 3 and flicker 31 Hz), implies therefore a reduction in the signal also in the mesopic condition, i.e., in transition from dark to light adaptation. The OPs extracted from filtering DA 3.0 and DA 10.0 traces are shown in the right panel of Figure 1 and appear to be noticeably reduced in the ACHM individual as compared with the healthy subject.

Mixed rod-cone responses elicited by light flashes of 3.0 cd·s·m^−2^ and 10.0 cd·s·m^−2^ luminance recorded by ffERG were considered for the analyses. Mean and standard deviation of implicit times and amplitudes for both a- and b-wave in each luminance level were extracted for each subject and compared between normal controls and achromats (Table 1). The results show an overall alteration in amplitude and implicit time of the conditions in the achromat group, with statistically significant differences for both a- and b-waves in both responses except the implicit time of the b-wave in DA 10.0. In both conditions, ACHM subjects show an average reduction in a- and b-wave of approximately 30% and prolongation of implicit time between 15 and 25%.

### 2.2. Complex Morlet Wavelet Transform

**Figure 2:**
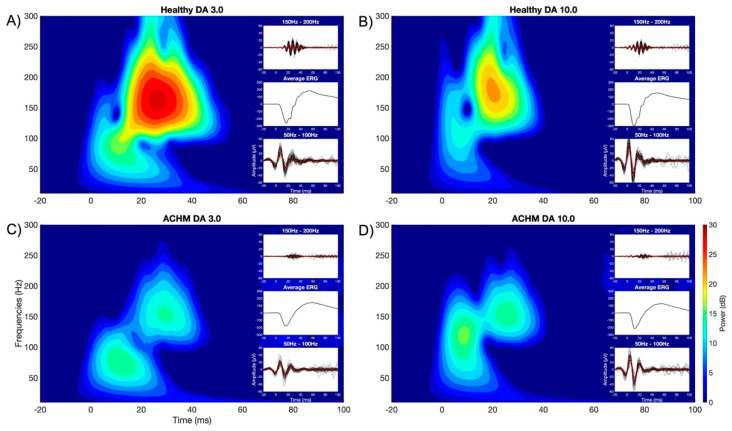
Time−frequency maps on the left indicate relative power (dB) under the DA 3.0 condition for healthy individuals (**A**) and ACHM (**C**). Time−frequency maps on the right indicate relative power (dB) under the DA 10.0 condition for healthy individuals (**B**) and ACHM (**D**). The traces depicted on the right side of each map represent the ffERG signal. From top to bottom, these traces represent: the ffERG trace filtered at 150–200 Hz (individual traces in black and the group average in red), the group average ERG trace, and the ERG trace filtered at 50–100 Hz (individual traces in black and the group average in red). To be noted in both DA 3.0 and DA 10.0 conditions, there is a considerable reduction in the signal in the filtered ffERG trace (150–200 Hz) of achromats compared to healthy controls, which is also reflected in the average ffERG.

Figure 2 shows the time-frequency maps obtained by convolving a complex Morlet wavelet with the acquired ffERG signals at different scotopic luminance intensities (DA 3.0 and 10.0). The top and bottom plots show the signal evolving in time (−20 to 100 ms) and frequency (10 to 300 Hz) in normal controls and achromats for DA 3.0 and DA 10.0 (left and right, respectively). The resulting scalograms were obtained by averaging the individual relative power (dB) normalized according to its baseline interval.

In all time-frequency plots, it is possible to observe two main clusters, one occurring earlier at a lower frequency (50–100 Hz) and the second occurring later at a higher frequency range (>150 Hz). In previous research, the presence of two oscillators at medium and high light exposure in scotopic conditions at different time-frequency windows have been reported [34,35]. These oscillators ascribed to the OPs occurring within the first 50 ms after the flash show two distinct frequency peaks, the first around 70 Hz and the second occurring at frequencies above 120 Hz. On the right of each time-frequency map, filtered traces of the signal from all the subjects are shown representing the time course of the OPs clusters (50–100 Hz and 150–200 Hz) and the average of the ffERG of the matching group. As can be seen on the top filtered trace (150–200 Hz), the OPs in individuals with ACHM appear to be delayed and have a notably reduced amplitude compared to the controls, while at lower frequencies the signal seems not to differ. This observation is confirmed by the results of the statistical cluster permutation test that compared the two groups at the different stimulus strengths shown in Figure 2. For both stimulations, a significant positive cluster (*p* < 0.05, one tail) has been found in the frequency band corresponding to the high OPs (>100 Hz). For the condition of DA 3.0, the significant cluster extends widely at the frequency band above 100 Hz and within 60 ms, while it is not significant at the time window corresponding to the lower frequency bands peaks (50–100 Hz). In the condition of DA 10.0, a significant positive cluster (*p* < 0.05, one tail) is limited only to the time window and frequency range corresponding to the OPs.

## 3. Discussion

The study aimed to characterize the ffERG signal in the time and frequency domains in a normal population and in individuals with ACHM using a complex Morlet wavelet transform. As reported in the previous literature [7,8,36], electrophysiological features of achromats show normal or near-normal rod-mediated responses when stimulated in dark adaptation, while functional cone signals are absent when stimulated in the light-adapted condition due to lack of functional cone photoreceptors. With the increase in stimulation strength in the dark-adapted eye from scotopic to mesopic, the light stimulus elicits mixed responses of cones and rods, favoring the appearance of OPs. In achromats, dysfunction of cones seems to yield alterations of the implicit time and amplitude of the a- and b-wave and the OPs under conditions of combined photoreceptor responses. In terms of amplitudes and implicit time comparisons (Table 1), our findings are in line with those reported by Zobor and co-workers (2017), in which it is shown that in the scotopic condition with a high luminance intensity (DA 3.0), the values of a- and b-wave were significantly altered compared to normal controls. While responses at low light stimulation are primarily mediated by rods and do not result in abnormal signals with increasing brightness, cone dysfunction largely affects the mesopic condition when mixed cone-rod responses are elicited [8].

**Figure 3:**
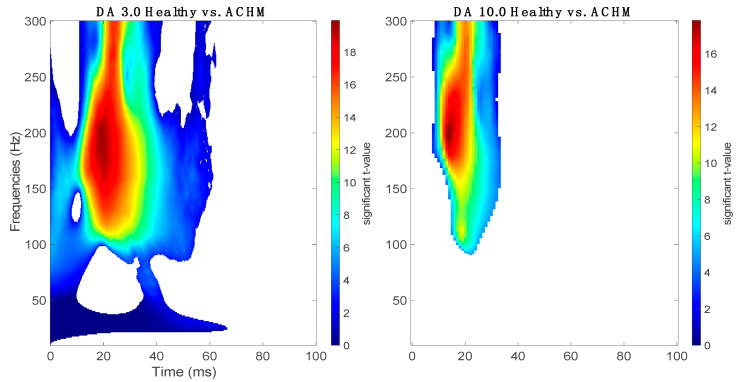
Statistical cluster-based permutation analysis: on the left, the comparison between normal and achromat groups shows a significant cluster (*p* < 0.05, one-tail) spread within 50 ms mainly at high frequencies at a luminance level of DA 3.0; on the right, the comparison between the healthy and the achromat groups shows a significant cluster (*p* < 0.05, one-tail) mainly focused on the second cluster of frequencies corresponding to the OPs at a luminance level of DA 10.0.

The main results of this study are the observations of the reduction in the relative power (dB) in time-frequency domains of the OPs using a continuous complex Morlet Wavelet transform (Figure 2). In the scotopic condition, previous studies investigating the time-frequency characterization of OPs claimed the presence of two frequency ranges within 50 ms after the onset of the stimulus, one between 70 and 100 Hz, and the second peaking at ~150 Hz [34,35]. Similarly, our results in the DA 3.0 and 10.0 conditions show the presence of two main frequency bands. By comparing the spectrograms of healthy controls and achromats, the cluster reaches statistical significance mainly in frequencies above 100 Hz, although the cluster in the DA 3.0 condition covers a wider range in frequencies and time (Figure 3). This evidence suggests that the absence of functional activity of cone photoreceptors may contribute significantly to the generation of high frequencies OPs in mixed rod-cone responses under dark adaptation following intense stimulation. The early component expressed at low frequencies is consistent with the study conducted by Dong and co-workers (2004), in which the authors suggest that the early subcomponents of OPs are mainly driven by the photoreceptors [29]. Thus, our findings reporting a significant difference primarily in the late component are a strong indicator that the major generator of the early component may be related to the rod function, and only to a small extent to cones.

To be noted in the spectrogram of healthy individuals is the presence of “holes” within a specific frequency band (Figure 2). These holes, also reported by Forte and co-workers (2008) as a drop in signal magnitude, appear early (between a-wave and OPs) around a frequency of 100 Hz and could suggest the presence of multiple oscillators interfering with each other in the ffERG signal [34]. These aforementioned holes seen in normal eyes and not in achromats may provide relevant information regarding the function of the photoreceptors in generating the OPs, so that interference between rods and cones occurs in higher frequency bands.

As discussed above, the late/high-frequency component of OPs is significantly reduced in the ACHM group, possibly due to the lack of cones modulation on the generation of the OPs. However, although notably attenuated, it is possible to observe OPs’ activity at high frequencies in achromats, possibly generated by rod activation alone. Concerning this observation, we can suggest several possible explanations.

Previous studies have shown that rods can make direct connections between cones and OFF cones bipolar cells [37,38]. In this respect, some interaction of excitatory and inhibitory circuitry, hypothesized as one of the generators of OPs, could be still achieved even with rods alone. Moreover, the current model of synaptic plasticity in animal models of ACHM does suggest a certain level of rewiring between rods and the inner retina [39]. The presented results are limited in providing or defining exact generators of the observed changes in OPs; however, they do indicate several extremely important findings regarding the clinical relevance of OPs. For instance, current therapeutic approaches successfully restored cone function in animal models [40,41]. However, first phases I/II clinical gene therapy trials in human ACHM only showed a very limited effect on the cone function restoration [42,43]. In human trials, changes in ffERG responses could not be observed, although all previously published ACHM animal models showed a clear improvement in ffERG responses upon gene augmentation [41]. However, results cannot be directly compared as all ACHM animal models were treated at early ages, while patients in the published ACHM gene therapeutic trials were aged 18 or older. Moreover, if there had been any effect on the retinal network visible in the ffERG in humans, amblyopic changes in adult achromats could additionally have caused the missing restoration of visual acuity. However, current strategies in functional evaluation in ACHM gene therapy could not reveal the actual cause for the unequal cone rescue in animals and humans to date. An optimal strategy of retinal functional evaluation would provide us with information on how many photoreceptors have been functionally restored, how they are connecting to the inner retina, how this information is processed on the level of the ganglion cell, and ultimately on the level of the visual cortex. Data from our study suggest that OPs can be an important marker of the information transfer from photoreceptors to the inner retina and an indirect biomarker for the number of functionally active cones. In this respect, OPs as a sign of retinal processing should be one of the key readouts in comprehending how the functional rescue of cones is projected to the retinal network.

## 4. Materials and Methods

### 4.1. Preparation and ffERG Recordings

The full-field electroretinography (ffERG) was performed using a ColorDome stimulator and the Espion E2 software (Diagnosys Ltd., Cambridge, UK) following ISCEV standards [11]. The analyses were conducted on 51 healthy participants (36 females and 15 males, mean age 34.61 ± 13.11 std) and 41 achromats (20 females and 21 males, 30.92 ± 14.13 std; 18 *CNGB3*-ACHM, 22 *CNGA3*-ACHM, 1 *PDE6C*-ACHM), and limited to dark-adapted 3.0 and 10.0 cd·s·m^−2^ (ISCEV DA (dark-adapted) 3.0 and 10.0, respectively) luminance levels, as they represent a good stimulation for retrieving combined rod-cone responses. The measurement was conducted on both eyes with dilated pupils (topical tropicamide (0.5%) and phenylephrine hydrochloride (2.5%), Mydriaticum Stulln, Pharma Stulln, Stulln, Germany) after 20 min of dark adaptation. Silver corneal fiber electrodes (DTL-electrode, Diagnosys Ltd., Dublin, Ireland) were positioned in the lower conjunctival bag, and gold cup reference and ground electrodes were placed with a paste at the ipsilateral temples and forehead, respectively. Each light stimulus was delivered at 1 Hz using a white-6500 K pulse against a white background of 0 cd/m^2^. A bandpass filter was applied after the recording with a bandwidth of 0.3–300 Hz. For each condition, five responses were recorded and averaged to yield a single trace. Subsequently, the resulting individual responses of both eyes were averaged together. A sample frequency of 5 kHz and 1 kHz were used to record DA 3.0 and DA 10.0, respectively.

### 4.2. ffERG Analysis

On the time domain, implicit time and amplitude of the scotopic a- and b-wave were extracted from the averaged signal over trials and channels of each subject. For the a-wave, the most negative trough after the onset of the flash was considered to extract the amplitude, while the first most positive peak following the negative trough of the a-wave was considered as the b-wave amplitude. The implicit time of both components was extracted from the flash onset to the respective peaks.

In order to localize changes in the frequencies over time, a complex Morlet wavelet was used to characterize the dynamics of the ffERG signal. The use of the Morlet wavelet was previously implemented for the analysis of the ffERG signal [34,35]. Since biological signals are assumed to be non-stationary, the employment of a Fourier transform (FT) alone to extract only the frequency is inappropriate. Conversely, the application of the Morlet wavelet helps to extract both temporal and frequency features of the ffERG components from the signal. A complex Morlet wavelet, defined as the product of a complex sine wave and a Gaussian, is described by the following formula [44]:

$$cmw=e^{i2\pi ft}e^{-t^{2}/2s^{2}}$$

in the first part of the equation defining the complex sine wave, *i* is the imaginary part ($i=\sqrt{1}$), *f* is the frequency of the wave and *t* is time, while in the second part *s* is the standard deviation in the formula for the Gaussian:

$$s=\frac{n}{2\pi f}$$

the parameter *n* represents the number of cycles of the wavelet. In order to define the right trade-off between temporal and frequency precision, a range of several cycles (from 3 to 7) that increases logarithmically has been created as a function of the frequencies considered for the analysis range (10–300 Hz) in which the ffERG components occur. By using this method, lower frequencies would have a better temporal resolution, while higher frequencies would have more cycles and better frequency resolution. The resulting time-frequency spectrogram for each subject was converted to the decibel (dB) scale through baseline division and log transformation. The analyses were performed using Matlab R2018b (Mathworks, Natick, MA, USA) and Fieldtrip Toolbox [45].

### 4.3. Statistical Analysis

In order to compare implicit times and amplitude of the ffERG a- and b-wave of both groups, the Shapiro–Wilk test was first applied to establish whether each dataset followed a normal distribution, and then the non-parametric Wilcoxon rank-sum test was performed to detect significance.

The effect of the Morlet wavelet transform between the two groups for each condition was identified by examining the difference in power with a paired t-test at each time point and each frequency. This previously described method [46] allows the simultaneous identification of clusters in time and frequency for which the results of the t-test were significant (*p* < 0.05, one tail). A random permutation of the conditions within the participants was subsequently performed and the test was repeated 1000 times. For each permutation, the t-values belonging to each cluster and having a *p*-value < 0.05 were totaled to estimate the distribution of the null hypothesis.

## Figures and Tables

**Table 1:** Mean and standard deviation of amplitudes and implicit times of a- and b-waves (DA 3.0 and DA 10.0). Asterisks (**) correspond to a *p*-value < 0.001.

		Normal Controls		ACHM	
		Amplitude (Mean ± Std)	Implicit Time (Mean ± Std)	Amplitude (Mean ± Std)	Implicit Time (Mean ± Std)
DA 3.0	a-wave	−264.8 ± 61.8	14.6 ± 1.08	−185 ± 50.9 **	16.20 ± 1.48 **
	b-wave	449.8 ± 107.49	48.4 ± 4.10	324.17 ± 93 **	53.33 ± 5.3 **
DA 10.0	a-wave	−302.57 ± 65.6	11.07 ± 0.9	−219.99 ± 60.7 **	12.35 ± 1 **
	b-wave	456.50 ± 116.47	47.38 ± 4.8	344.96 ± 91.8 **	49.14 ± 4.6

## Data Availability

Raw data were generated at the University Eye Hospital in Tübingen. Derived data supporting the findings of this study are available from the corresponding author G.R. on request.
